# Dynamic Monitoring Reveals Motor Task Characteristics in Prehistoric Technical Gestures

**DOI:** 10.1371/journal.pone.0134570

**Published:** 2015-08-18

**Authors:** Johannes Pfleging, Marius Stücheli, Radu Iovita, Jonas Buchli

**Affiliations:** 1 Agile and Dexterous Robotics Lab, ETH Zürich, Zürich, Switzerland; 2 Product Development Group Zurich, ETH Zürich, Zürich, Switzerland; 3 MONREPOS Archaeological Research Centre and Museum, RGZM, Neuwied, Germany; University of Oxford, UNITED KINGDOM

## Abstract

Reconstructing ancient technical gestures associated with simple tool actions is crucial for understanding the co-evolution of the human forelimb and its associated control-related cognitive functions on the one hand, and of the human technological arsenal on the other hand. Although the topic of gesture is an old one in Paleolithic archaeology and in anthropology in general, very few studies have taken advantage of the new technologies from the science of kinematics in order to improve replicative experimental protocols. Recent work in paleoanthropology has shown the potential of monitored replicative experiments to reconstruct tool-use-related motions through the study of fossil bones, but so far comparatively little has been done to examine the dynamics of the tool itself. In this paper, we demonstrate that we can statistically differentiate gestures used in a simple scraping task through dynamic monitoring. Dynamics combines kinematics (position, orientation, and speed) with contact mechanical parameters (force and torque). Taken together, these parameters are important because they play a role in the formation of a visible archaeological signature, use-wear. We present our new affordable, yet precise methodology for measuring the dynamics of a simple hide-scraping task, carried out using a pull-to (PT) and a push-away (PA) gesture. A strain gage force sensor combined with a visual tag tracking system records force, torque, as well as position and orientation of hafted flint stone tools. The set-up allows switching between two tool configurations, one with distal and the other one with perpendicular hafting of the scrapers, to allow for ethnographically plausible reconstructions. The data show statistically significant differences between the two gestures: scraping away from the body (PA) generates higher shearing forces, but requires greater hand torque. Moreover, most benchmarks associated with the PA gesture are more highly variable than in the PT gesture. These results demonstrate that different gestures used in ‘common’ prehistoric tasks can be distinguished quantitatively based on their dynamic parameters. Future research needs to assess our ability to reconstruct these parameters from observed use-wear patterns.

## 1 Introduction

The use of stone tools is one of the defining characteristics of our species, but has so far in human evolution played a rather secondary explanatory role to that occupied by their manufacture. By this we mean that, although ancient hominins most certainly made tools in order to use them, most of the debates in archaeology relating to stone tools refer to various aspects of tool production and the morphology thought to result from manufacture. One of the obstacles in developing a complex theory of tool use as a decisive behavioral factor in human evolutionary history is the fact that, once invented 3.3-2.6 Mya, the earliest (stone) tools remain morphologically unchanged for millions of years, while anatomy undergoes major changes. This apparent divergence gives the impression that stone tools were largely neutral to post-Oldowan hominin adaptation until much later, when more complex technologies emerged, such as distance weapons. However, recent work in paleoanthropology has shown that the anatomical prerequisites for complex manipulation of hand-held tools may have evolved very early [1]. This research was made possible by new methods which can track and model the loads involved in tool making and tool use result in plastic responses of the skeleton which are visible in the fossil material [1–4]. In this way, it is possible to reconstruct the history of precision and power grips, of throwing and of other habitual tasks. However, as informative as such studies are, they provide only half of the picture. The act of operating a tool to manipulate the environment leaves traces at both ends, on the body that transfers the energy and, of course, on the tool itself, in the form of wear and damage. Moreover, stone tools with use-wear traces are much more frequently preserved than fossils. Therefore, in order to deliver a fine-grained historical account of tool use that is relevant to biological evolution, it is necessary to devote time and effort to studying the effect of dynamics on stone tool wear.

**Fig 1:**
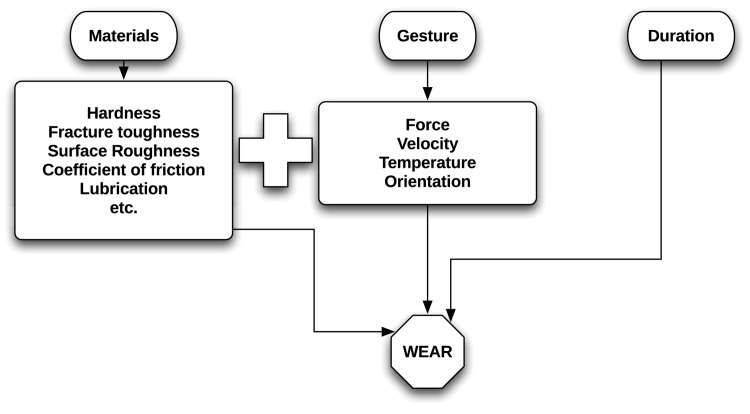
The contribution of gesture in the formation of tool use wear. Wear due to repeated use of a tool to work a second material is dependent on the physical properties of the contact materials, the duration of the action [33–35], as well as on the dynamics [36–38], which, in the context of tool use, are dictated by the chosen gesture.

In this context, the reconstruction of the movement involved in the operation of tools becomes central to a holistic understanding of the coevolution of technology and biology. A first step in this direction is to investigate whether or not there is sufficient dynamic variation in different technical gestures used in the carrying out of specific tasks (such as hide-scraping, butchery, etc.) to expect corresponding variation in wear formation. By technical gesture [5–8], we mean the culturally-mediated amalgam of body and tool movements that comprise a technical action, such as scraping or cutting. We use the term dynamics to denote the combination of kinematics (position, orientation, and speed) with mechanical contact parameters (force and torque). As shown in Fig 1, tribological wear is determined by a variety of factors, one of the most important of which is the dynamics (see also [9]). In tool use, the latter is determined by gesture, and yet, most use-wear experiments are carried out with some degree of control on the materials and the duration of contact, but without recording the dynamics (but see [10] for a recent attempt). Thus, only a rough reconstruction of the gesture is currently possible (for example, the direction of tool motion ‘transversal’ or ‘longitudinal’ to the working edge [9, 11–16] and sometimes the angle and hafting arrangement [17, 18]). Our paper sets out to fill in this gap by proposing a methodology for monitoring task dynamics and testing its ability to statistically distinguish gestures using their associated parameters. The second step in this research program will be to search for a secure, preferably quantitative link between observable surface modifications associated with wear (polishes, striations, surface roughness, etc.) and the dynamic parameters corresponding to different gestures. This is the subject of a future paper.

## 2 Materials and Methods

### 2.1 General description of the task and gestures

We focus on one simple hide-processing task carried out with hafted endscrapers using two different gestures: pull-to (PT) and push-away (PA). These tools are associated with hide processing in both ethnographic [18–22] and archaeological contexts (e.g., [23–25]) and a comparably large amount of information is known about the types of movements involved, as well as the hafting arrangements and general conditions of use (although see [26] for a different view). Moreover, the range of motion for these two gestures is relatively constrained (in a mostly linear direction perpendicular to the tool edge), compared with actions such as ‘cutting’ (see [27]). This allows us to test the hypothesis that dynamic parameters (e.g., speed, force, torque, etc.) associated with the contact conditions at the tool tip can help distinguish the gestures, even if the range of motion is small. For these reasons, endscraper hide-processing makes a good proof-of-concept case-study for our methodology.

Before going further, it is worth dwelling upon the issue of plausibility, which is one that frequently comes up in archaeological experiments. More specifically, the question is: How realistically does an experiment reconstruct particular prehistoric tasks? The ethnographic record teaches us, if nothing else, that our researcher’s imagination is simply too limited to account for the seemingly countless ways of performing simple tasks (for the most recent review see [28]). However, the goal of an experiment should be to simplify, model, and understand, not to reconstruct the study object (in this case, past actions) in its entirety. For this reason, a certain fine balance between plausibility and simplicity has to be struck by the experimenter. In our study, whose aim is to test the ability of our measurement configuration to record and characterize gestures, we have had to limit the number of possibilities involved in positioning the hide, hafting the tools, and choosing the gestures. In future studies, we anticipate greatly expanding the allowed variation in these variables.

Although hide scraping can also be performed with handheld flakes, we used hafted tools in order to facilitate the measurements. Following some ethnographic examples (e.g., [29]), the perpendicularly hafted tool was used for the PT gesture and the distally-hafted tool for the PA gesture (e.g., [20]). The perpendicularly-hafted scraper can be used for a PA gesture, too, by turning it around by 180 deg. Although this mode of working is known from some ethnographic examples [18, 29], it is usually associated with holding the hide in the hand, thus introducing extra variables (such as variable angles) elsewhere in the experimental design. Moreover, while using different hafting arrangements may appear to introduce unnecessary variables, the geometry of the particular arrangements we have chosen is most similar to the handheld case (perpendicular vs. in continuation of the arm).

**Fig 2:**
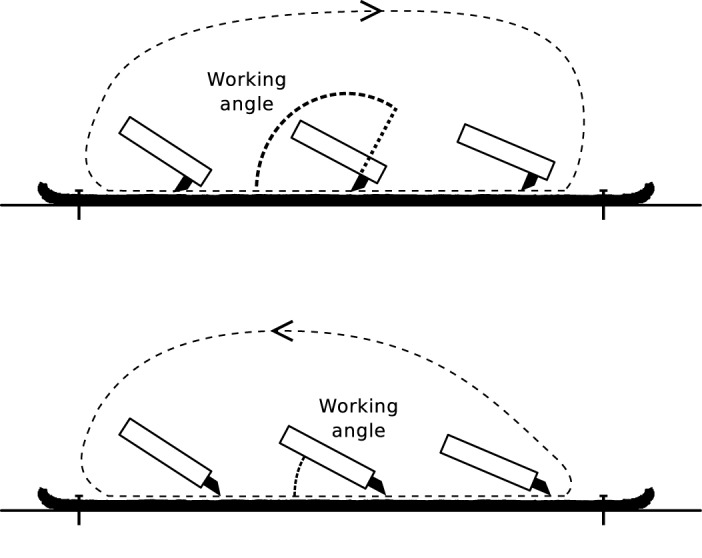
Tool path of one cycle for the PT gesture (top) and the PA gesture (bottom). The tool is depicted from side view for three time instances. The angle between workpiece and plane surface of the scraper is denoted as working angle. The user operates the tool standing at the left hand side.

**Fig 3:**
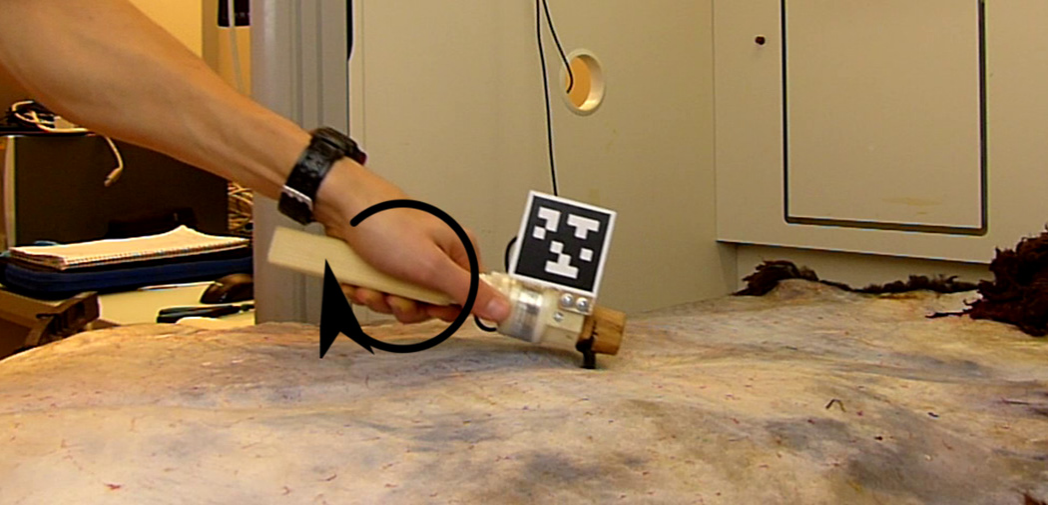
Illustration of tool handling during the PT gesture on fresh hide. The subject grasps the tool at the shaft. Hand torque is required in order to apply force to the tool tip (illustrated as black arrow).

The subjects were shown and explained how to perform the task. They were free in interpretation of the instructions. This means that no tight control of the subjects over kinematic or dynamic aspects was exerted. The stated goal was “the removal of fat and flesh from the hide”. The body posture necessary to carry out the task was left to individual choice. For both tasks the subject was operating from the left hand side of the workspace, as depicted in Fig 2. The subject held the tool in a hammer-like grasp as depicted in Fig 3. Furthermore, subjects were encouraged to vary the length of the stroke phase and magnitude of force in different experiments. Thereby we increased the variability in the dataset, which achieves two goals: first, it raises the bar higher for our measurement configuration and avoids trivial results; and second, it allows us to present upper and lower bounds for the general task parameters.

**Fig 4:**
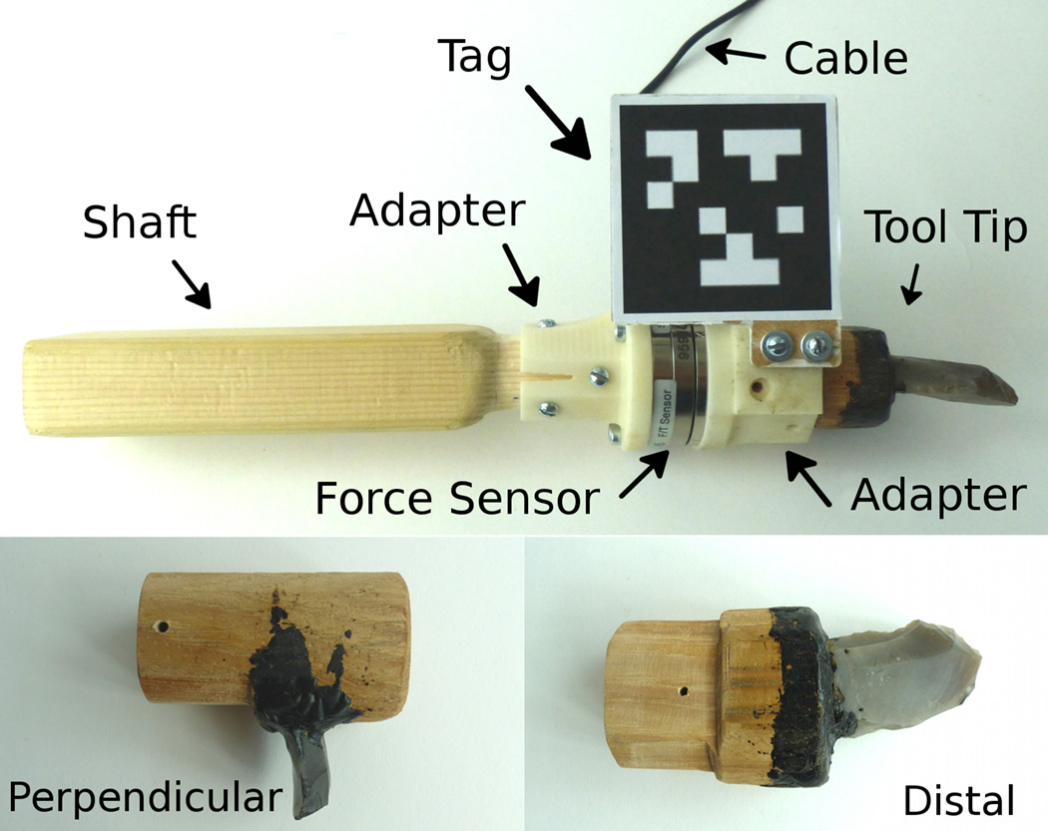
The tool (top) and exchangeable tool tips (bottom). Parts of the sensor system is integrated in the tool. Not shown is the protection film, which was wrapped around the force sensor during the experiments.

The task dynamics can be described in terms of the tool trajectory for one cycle (Fig 2). The part of the cycle where the tool tip is in contact to the workpiece is what we call the “stroke phase”. Correspondingly, we call the remaining part of the cycle “flight phase”. Scraping with the PT gesture is performed with the perpendicularly hafted tool (Fig 4) with the ventral (plane) surface of the scraper leading in direction of motion. During the stroke phase the tool is pulled towards the user following a straight line. The approximate tool orientation is as depicted in 2, where the working angle is larger than 90 deg. When using the PA gesture, the distally hafted tool is used with the dorsal (convex) tip surface pointing upwards. Contrary to the PT gesture, the tool is pushed away from the user. The working angle is lower than 90 deg

### 2.2 Materials

The tool tips/bits used in the experiments were ethnographically and archaeologically plausible replicas of stone endscrapers. Flint flakes were knapped by one of us (RI) into scrapers and hafted using birch tar either distally along the axis of the haft or perpendicular to it, in the manner described by [19, 20, 30] for 20th century Ethiopian and Alaskan hide-scrapers. Sizes of the scrapers in length are 50 mm for perpendicular hafting and 70 mm for distal hafting. The bit is convex in plan view and has a plano-convex lateral cross-section. The shaft was cut and rasped in order to fit into the adapter mount.

The workpiece consisted of the hide of a young sheep, which was collected one day after death from an abattoir (Fleisch + Wurstproduktion Braunwalder Wohlen). The hide was first used fresh for 13 experimental sets, after which it was dried for six days under the fume hood at ambient temperature and used for a further 13 experimental sets.

### 2.3 Recording Set-up

**Fig 5:**
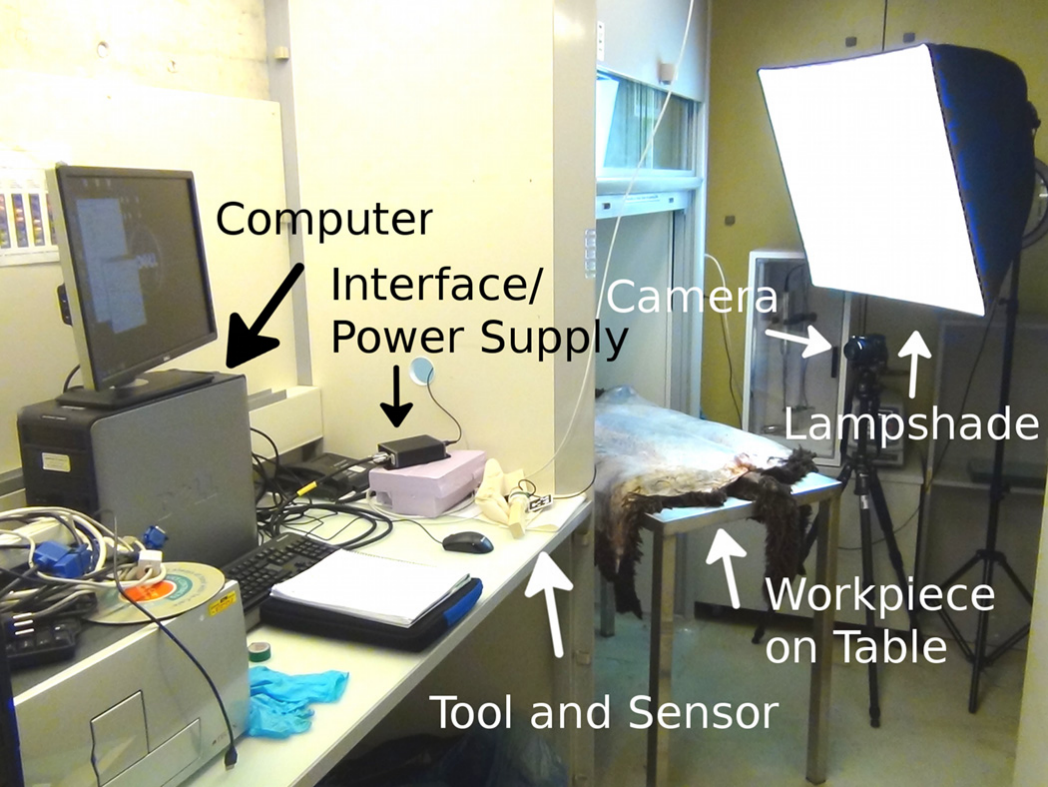
The Experimental Set-up in the lab. The workpiece depicted is fresh sheep hide. The table is partly arranged under a fume hood.

The experiments were performed in the lab (Fig 5). The workpiece was fixed tightly with nails onto a wooden plate with the wooly side facing down. This may appear to be unrealistic, yet it is commonly done using sticks and a flat ground surface in ethnographic cases [18, 29]. The plate was fixed on a metal table of 1.2 m in height by screw clamps. At the long edge of the table camera and lighting were placed on tripods. The camera was positioned at a distance of 0.4 m to the table and the optical axis horizontally and perpendicular to the long edge. In order to adjust the camera orientation we used a level. Two 85 W neon lights inside a lampshade were arranged behind and slightly above the camera and the lampshade was directed onto the hide to improve image quality. The subject stood at the short edge of the table during the experiments.

### 2.4 Measuring Configuration

The central part of the measurement system is the tool as depicted in Fig 4. The force sensor and the tag are attached to the haft and also linked to peripheral systems for data recording. The wooden shaft is connected to the plane side of the sensor via an adapter made of ABS plastic and screws. The leading side of the sensor is connected to the leading adapter which serves as holder for the tool tips. The tool tips fit into the adapter mount and are locked in place by screws. Additionally a square tag of 58 mm side length is fixed laterally to the adapter with the plane parallel to the long axis of the shaft. The tag is part of the AprilTags tracking system [31]. It was printed and placed on a wooden board. The choice of size is a trade-off between accuracy of the tracking system and limitation of tool handling. A soft protection film is wrapped around the sensor and fastened by plastic straps.

The force sensor (ATI Mini45) has a diameter of 45mm, a height of 15.7 mm and weighs 92 g. It is connected through a cable to the Interface Board/ Power Supply which in turn is connected to the data acquisition (DAQ) card (National Instruments PCI-6220 M 16-bit) of the PC. Proprietary software of ATI converts uncalibrated signals of the sensor to calibrated values. The sensor measures all three components of force and torque which are applied at the leading side of the sensor. Range and performance of the sensor are listed in Table 1. The range is sufficient to cover the strength of the human arm.

**Table 1:** Performance of the ATI Mini45 6-axis force sensor in combination with DAQ card. The sensor was calibrated by the manufacturer to the largest range available. The Measurement Uncertainty values are the maximum amount of error for each axis expressed as a percentage of its full-scale load.

	Range	Resolution	Meas. Uncertainty
*F*_*x*_	± 580 N	1 / 16 N	1.5%
*F*_*y*_	± 580 N	1/16 N	1.25%
*F*_*z*_	± 1160 N	1/16 N	1.0%
*T*_*x*_	± 20 Nm	1/752 Nm	1.25%
*T*_*y*_	± 20 Nm	1/752 Nm	1.75%
*T*_*z*_	± 20 Nm	1/504 Nm	1.0%

The position sensor consists of mid-price digital video camera (Panasonic Lumix DMC-FZ200) and the tag on the tool. Videos of the tool are recorded during the experiments. It is important that the printed side of the tag always be in field of view of the camera. At post processing we analyzed the videos using a C++ implementation (http://people.csail.mit.edu/kaess/apriltags/) of AprilTags. This software identifies the tag on the images and determines on the basis of tag size and camera intrinsics the position and orientation relative to the camera [31]. The information comprised in the 2D code on the tag is irrelevant for our application. We used the camera at resolution of 1280 × 720 pixels and at nominal focal length of 25mm. The camera has rolling shutter technique and it was calibrated using a camera calibration toolbox for Matlab (http://www.vision.caltech.edu/bouguetj/calib_doc/). For both, the camera and DAQ card we used sample frequencies of 100 Hz.

In order to establish the accuracy of the position and angular measurements in our system, we measured the positions simultaneously with our system and an OptiTrack (5 cameras: P13.FS.LENS0013, resolution: 1280 × 1024) motion capture system. The OptiTrack has an accuracy of 1mm. For our purpose we considered the Optitrack system as the gold standard for measurement. We calculated the error between the Optitrack data and our measurements and found a mean of 4.3 mm and a standard deviation of 3.2 mm of the position. Comparison of the orientation showed a mean error of 1.83 deg and a standard deviation of 1.77 deg. Both are for distances of the camera to the tag between 0.8 m to 1.2 m.

### 2.5 Experimental Protocol

1. Start recording of video camera and DAQ card of the PC.
2. Generate synchronization beat: In order to be able to synchronize measurement data of force and position sensor, we gently hit the tool on a hard object which is located within the workspace of the set-up. In doing so, we can identify that instant in time as a small peak in the plots of the data.
3. Perform task. The subject performs several cycles of one predefined task according to the instructions. The subjects had to ensure that the tool was kept within the workspace.
4. Stop recording of video and DAQ card of the PC.
5. Post processing of the video files with AprilTags.

After the post processing we obtained two comma-separated-values (csv) files containing the raw measurement data of the force and position sensors (see S1 Dataset). In a second processing step we used Matlab to do synchronization, coordinate transformation, statistical analysis and visualization of the measurement data.

Overall we generated an exploratory set of experiments with controlled variations in tool, task, workpiece, subject and user dependent variation in force and stroke phase. The maximum duration of the experiments was limited to 110 s in order to facilitate the data processing. Eight out of 23 experiments were performed with the non-dominant hand. For the statistical analysis we used a total number of 23 out of 26 experiments of which 13 used the PT and 10 the PA gesture. Three experiments were discarded because of errors of the measurement configuration during recording. S1 Table contains a summary of the data and statistics.

The subjects were recruited among staff and students of ETH Zürich. The subjects gave verbal informed consent. Thus, consent was documented by their participation in the study. No separate documentation of the consent was taken. The authors administered the study. In accordance with the ethics regulations of ETH Zürich no approval nor any formal waiver was required for this study.

### 2.6 Definition of Frames

**Fig 6:**
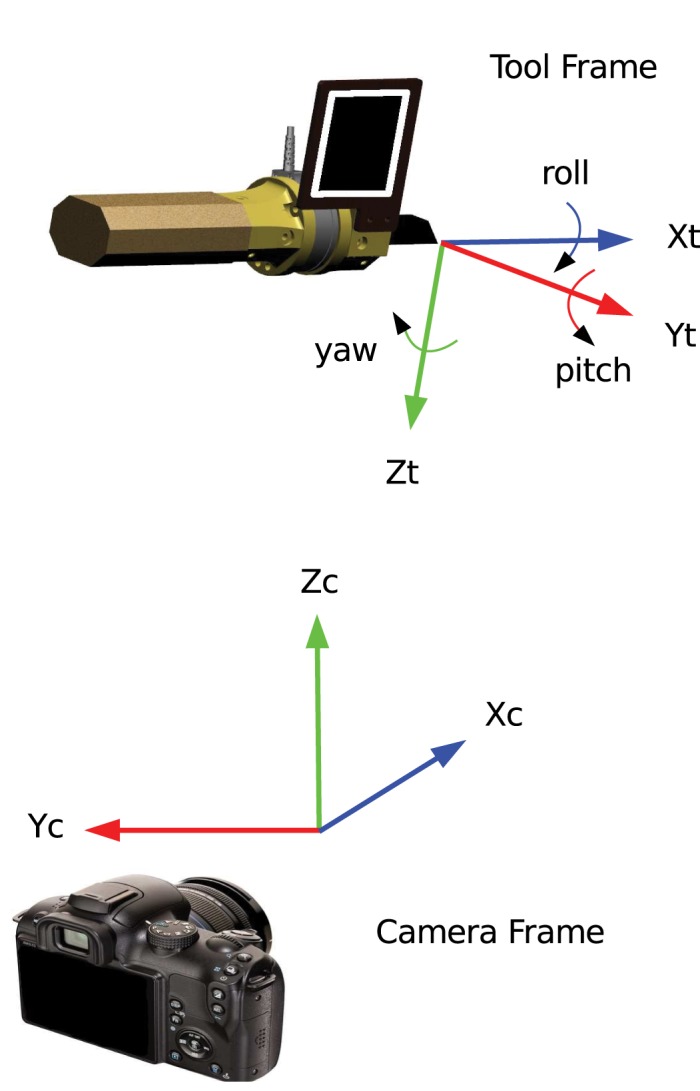
Definition of frames. The camera frame is fixed in space, whereas the tool frame is attached to the tool and moves with the tool. The position of the tool is described relative to the camera frame and the orientation relative to the reference orientation (see Eq 1).

We defined two right-handed Cartesian coordinate systems called camera frame and tool frame according to Fig 6. Technically, the camera frame is fixed to the camera which in turn is fixed relative to the workspace. Its origin is located at the center of the sensor of the camera. The *z* axis points upwards and parallel to the field of gravity so that the *x*-*y* plane corresponds to the plane of the hide. The camera frame is used to describe the position of the tool tip (*x*, *y*, *z*) relative to the camera as well as the force applied to the hide ($F_{x}^{c}$, $F_{y}^{c}$, $F_{z}^{c}$). Therefore, when the subject moves the tool up or down, it is moving in the *z* direction. When the subject moves the tool horizontally, it is moving either in the *x* or *y* direction or in a combination of both. The tool frame is attached to the tool with the origin fixed to its tip. The *x* axis is directed distal to and pointing away from the shaft and the *z* axis is oriented parallel to the tag. We use the tool frame to describe the force ($F_{x}^{t}$, $F_{y}^{t}$, $F_{z}^{t}$) and the torque ($T_{x}^{t}$, $T_{y}^{t}$, $T_{z}^{t}$) applied to the tool. The orientation of the tool is given in local Euler angles (X-*roll*, Y-*pitch*, Z-*yaw*), which describe the rotational transformation from camera frame to tool frame. For *roll* = *pitch* = *yaw* = 0, the tool frame is at reference orientation, which we define as:

$$\begin{array}{ccc} x_{t} & = & -y_{c},\\ y_{t} & = & -x_{c},\\ z_{t} & = & -z_{c}. \end{array}$$ (1)

Inherently, the AprilTags software only gives the position of the center of the tag. In order to determine the position of the tool tip, the geometry of the tool must be known. This was calculated using the measured distance from the center of the tag to the tool tip. Such measured kinematic quantities are further used to map forces and torques from one coordinate frame to another as required.

### 2.7 Statistical Analysis

The differences between the gestures were evaluated using a statistical analysis of the different benchmarks. Benchmarks are defined as the time-averaged values of the raw variables measured during the experiments.

For the measurement data of force and torque we took the average (< … >) over time *t* during all stroke phases as:

$$\begin{array}{c} <f\left(t\right)>=\frac{1}{N}\sum_{1}^{N}\frac{1}{\tau_{i}}\int_{\tau_{i}}f\left(t\right)dt, \end{array}$$ (2)

where *f*(*t*) is either force or torque, *τ*_*i*_ the time interval of the stroke phase *i*, and *N* the total number of stroke phases of the experiment, i.e. the number of repetitions. According to this definition we derived the following benchmarks:

- *Force* in the tool and in the camera frame, where the components are average values:
  $$\begin{array}{c} \bar{F^{t}}=(\begin{array}{c} <F_{x}^{t}>\\ <F_{y}^{t}>\\ <F_{z}^{t}> \end{array})\;,\;\bar{F^{c}}=(\begin{array}{c} <F_{x}^{c}>\\ <F_{y}^{c}>\\ <F_{z}^{c}> \end{array}) \end{array}$$ (3)
- *Shear force*, which we define as the component of force in the *x*-*y* plane of the camera frame:
  $$\begin{array}{c} \bar{F_{s}}=<\sqrt{F_{x}^{c}{\left(t\right)}^{2}+F_{y}^{c}{\left(t\right)}^{2}}> \end{array}$$ (4)
  Mechanically, *F*_*s*_ is the component of the force which is effectively responsible for the removal of material from the workpiece. The larger *F*_*s*_, the more material can be sheared off per stroke, and the greater the maximum strength of tissue bonds which can be cut by the tool.
- *Torque* in the tool frame consisting of three components:
  $$\begin{array}{c} \bar{T^{t}}=(\begin{array}{c} <T_{x}^{t}>\\ <T_{y}^{t}>\\ <T_{z}^{t}> \end{array}) \end{array}$$ (5)
  It is the average torque that is applied to the tool at the position of the sensor.
- *Hand torque*, which we define as the torque which is applied to the shaft through the hand of the user (see Fig 3). We determine it according to the following equation:
  $$\begin{array}{c} {\bar{T}}_{h}=<\sqrt{T_{hx}^{2}+T_{hy}^{2}+T_{hz}^{2}}> \end{array}$$ (6)
  Each component is determined by the torque in tool frame (*T* ^*t*^) and the distance from the sensor to the position on the shaft where the user grasps the tool (*l*_*sh*_):
  $$\begin{array}{ccc} T_{hx} & = & T_{x}^{t} \end{array}$$ (7)
  $$\begin{array}{ccc} T_{hy} & = & T_{y}^{t}+F_{z}^{t}▪l_{sh} \end{array}$$ (8)
  $$\begin{array}{ccc} T_{hz} & = & T_{z}^{t}+F_{y}^{t}▪l_{sh}. \end{array}$$ (9)
  We assume that the position of the grasp is shifted about *l*_*sh*_ = 10 cm in −*x* direction from the sensor position for both hafting types. The position of the grasp is an approximation of the idealized point where the user applies force and torque to the tool.

The following quantities were derived from the measurement data of position and orientation. The average (< … >) is defined for each benchmark individually.

- We define *stroke length* as the distance between the starting point and the end point of the stroke phase:
  $$\begin{array}{c} \bar{\Delta X}=(\begin{array}{c} <\Delta x>\\ <\Delta y>\\ <\Delta z> \end{array})=\frac{1}{N}\sum_{i=1}^{N}(X\left(\tau_{i}^{end}\right)-X\left(\tau_{i}^{start}\right)), \end{array}$$ (10)
  where $X=\left(\begin{array}{c} x\\ y\\ z \end{array}\right)$ is the position vector of the tool tip in camera frame and $\tau_{i}^{start}$ and $\tau_{i}^{end}$ are the time instances at the beginning and end of stroke phase *i*.
- *Total stroke length*, which we define as the combined motion in *x*-*y* plane.
  $$\begin{array}{c} \bar{\Delta X_{tot}}=\frac{1}{N}\sum_{i=1}^{N}\sqrt{{\left(x\left(\tau_{i}^{end}\right)-x\left(\tau_{i}^{start}\right)\right)}^{2}+{\left(y\left(\tau_{i}^{end}\right)-y\left(\tau_{i}^{start}\right)\right)}^{2}} \end{array}$$ (11)
- Finally, we calculate the *variance* of the orientation, $Var\left(\Phi \right)=\left(\begin{array}{c} Var(roll)\\ Var(pitch)\\ Var(yaw) \end{array}\right)$ using the following well-known formula for the variance of *yaw*, *pitch*, *roll* respectively:
  $$\begin{array}{c} Var(roll)=\frac{1}{n-1}\sum_{k=1}^{n}{\left(roll_{k}-\bar{roll}\right)}^{2}, \end{array}$$ (12)
  where *roll*_*k*_ is the *k*th time instance, *n* the total number of time instances of all stroke phases, and $\bar{roll}=\frac{1}{n}\sum_{k=1}^{n}x_{i}$ the mean over all time instances of all stroke phases. The same applies for *Var*(*pitch*) and *Var*(*yaw*) analogously.

## 3 Results and Discussion

The experimental set-up allows us to measure the force and the torque applied between stone tip and shaft as well as the position and orientation of the tool over time. Using computer software (Matlab) we are able to process and illustrate the data in different reference frames, i.e. from different perspectives (Section 3.1). Most importantly, statistical analysis identifies the parameters that are unique to each gesture (Section 3.2).

### 3.1 Characterizing the task

**Fig 7:**
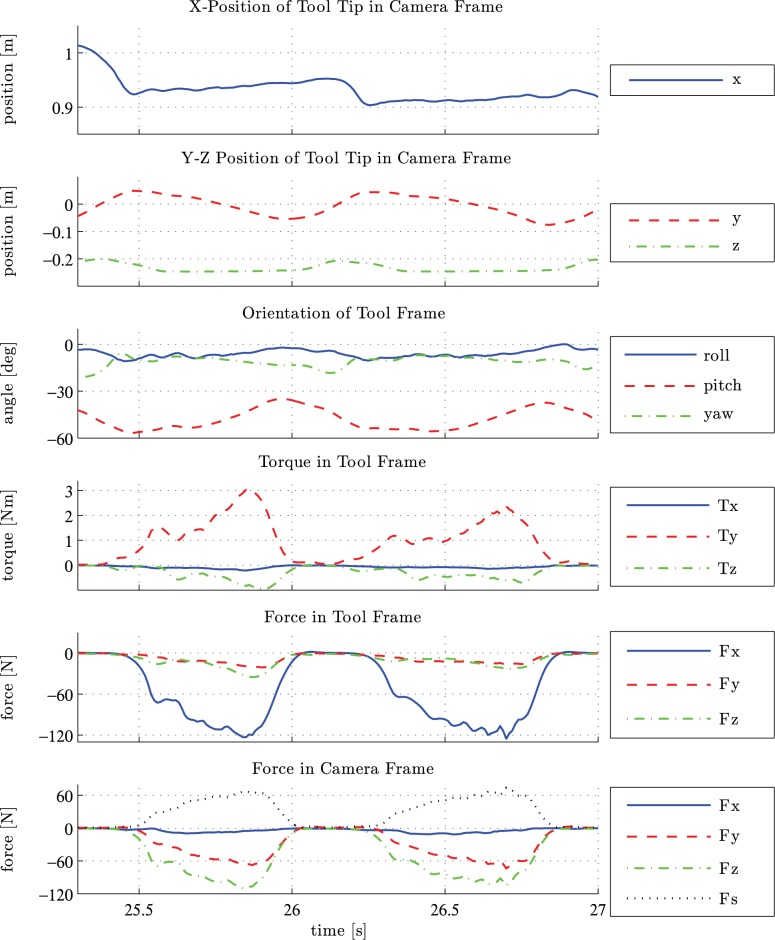
Plots of position, orientation, torque and force over time. The data shows two cycles of the PA gesture. Axes are defined in Fig 6. The position represents the location of the tip of the tool relative to the camera. The *z* position is plotted in a different scale than *x* and *y* position due to a lower range. The orientation of the tool is represented by *roll*-*pitch*-*yaw* local Euler angles. The data for torque shows the magnitude experienced by the tool at the location of the sensor. The Force experienced by the tool is depicted in tool frame and the force on the workpiece is depicted in camera frame. Additionally, shear force *F*_*s*_ is shown in camera frame.

**Fig 8:**
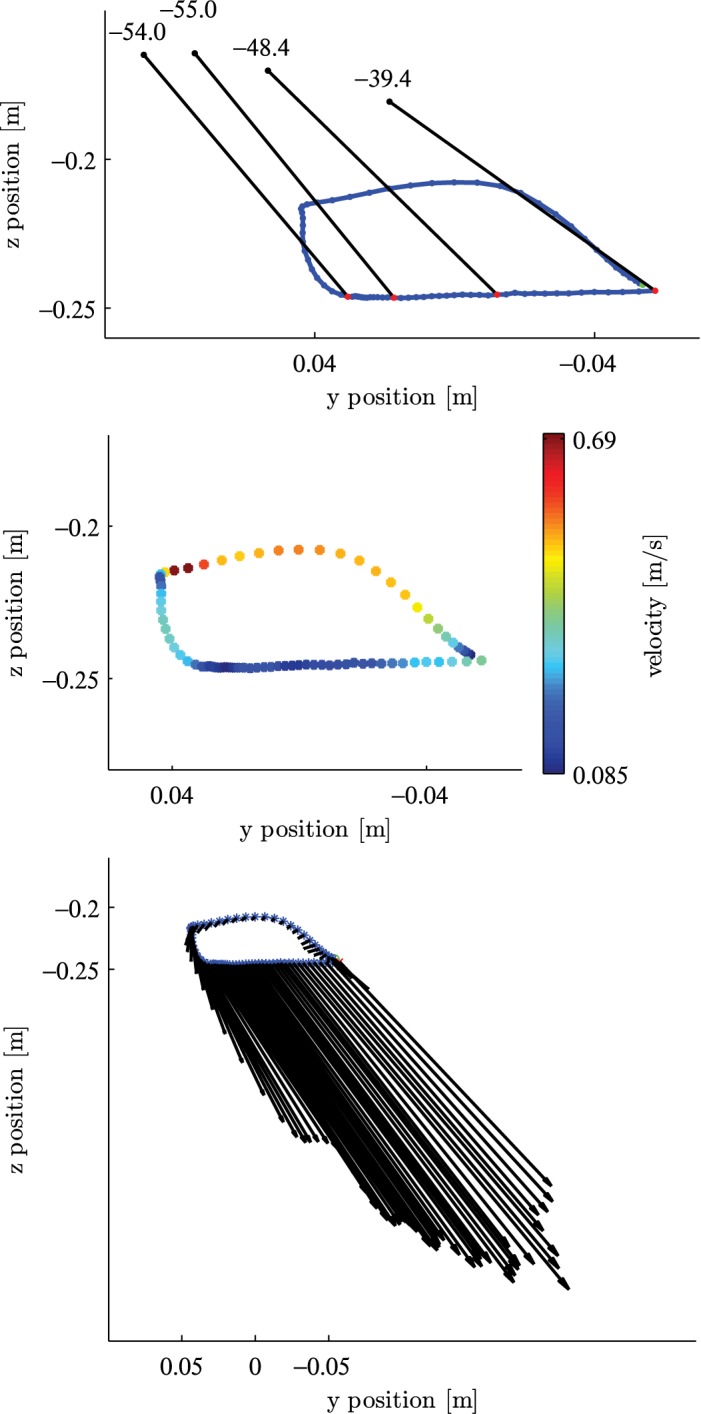
Trajectory of one cycle of the PA gesture. The data is shown in *y* and *z* axis, whereas the *y* axis points leftwards. Additionally, orientation in degree (top), velocity (middle) and force vectors (bottom) are illustrated. Motion is in counterclockwise direction.

Figs 7 and 8 showcase the measurement data for one representative experiment (exp24 in S1 Table) captured in several graphs. Here the PA gesture was used on a fresh hide and the subject held the tool with the dominant hand. The time interval depicted is between 25.3 s to 27 s after the synchronization beat.

Fig 7 shows the data sets of the dynamic parameters over time. Two tool cycles can be identified, each characterized by two successive minima of the *y* position. During elevated values of the *z* position up to −0.2 m the tool is in the flight phase (i.e. not touching the hide). This can also be seen by zero values for force and torque. The tool is swiftly returned to the starting point of the next stroke phase, which can be seen in the steep rise of *y* position. The stroke phase can be identified by constant *z* position and non-zero values for force and torque. The *y* position decreases slowly from maximum to the following minimum. Within one cycle, the orientation of the tool varies mainly in the *yaw* angle (equivalent to the working angle). Furthermore, the slight variation in *x* position indicates that the tool trajectory is not limited to the *y*-*z* plane. The starting point of the stroke phase of the first cycle is shifted about 0.02 m in the *x* direction compared to the starting point of the second cycle.

In Fig 8 only the second tool cycle of the observed time interval is considered. Tool trajectory is combined with orientation, velocity and forces in the *y*-*z* plane.

The upper plot shows the numeric values of the pitch angle, which decrease considerably during the stroke phase. The increase in distance between equally spaced time instances shows that the velocity increases towards the leftmost extreme point.

The middle plot shows the tool trajectory combined with the tool velocity. The velocity varies between 0.085 ms^−1^ and 0.69 ms^−1^. The maximum velocity is reached at the end of the flight phase when the tool touches the workpiece. During the stroke phase, the velocity reaches a local peak of approximately 0.4 ms^−1^ at the end of the stroke phase.

The combination of the trajectory and force vectors, illustrated as arrows is depicted in the lower plot. The amount of force increases during the stroke phase until reaching 2/3 of the stroke length and decreases slightly towards the end of the stroke phase. Another interesting aspect is the transition from the flight phase to the stroke phase at the left turning point of the tool path. The fact that the tool is moving downwards while the force is increasing indicates that the workpiece gives way until the pressure is high enough to allow the removal of material. This behavior was observed for both dry and fresh hide but more markedly for the fresh hide.

### 3.2 Differentiating the gestures

**Fig 9:**
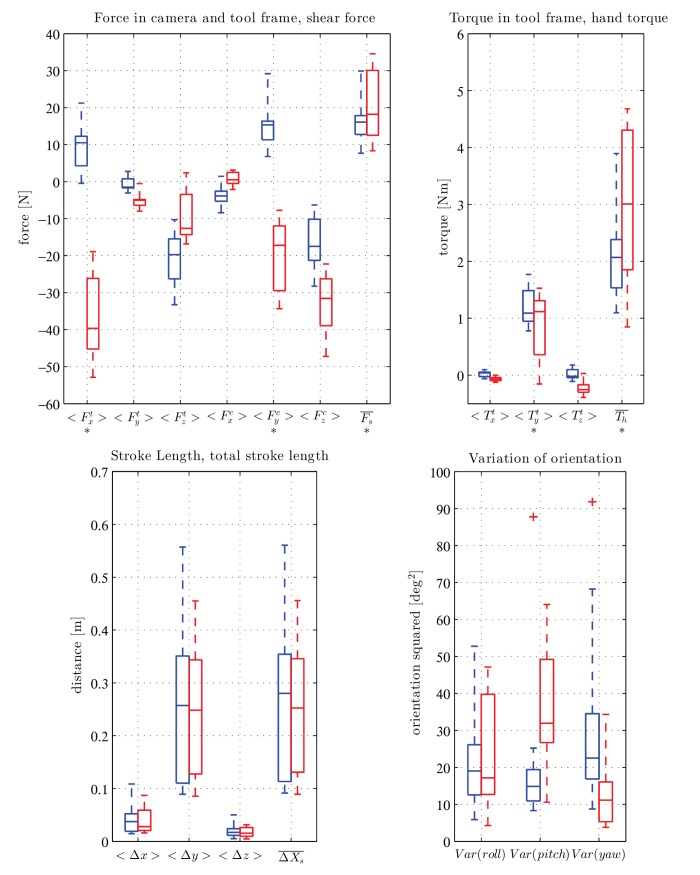
Box plots of benchmarks to characterize the tasks. PT is in blue (left) and PA is in red (right). The benchmarks are average values as defined in Section 2.7. We evaluated the benchmarks for a total of 23 experiments (13 PT and 10 PA, see S1 Table). The bar inside the box indicates the median. Edges of the square show the 25th and 75th percentiles. Whiskers are indicated by the dotted lines and outliers by a red cross. Stars below the labels on the *x* axis indicate significant difference in variability between PT and PA gesture.

The main aim of the study was to see if different gestures can be quantitatively separated using dynamic parameters recorded with a simple and relatively inexpensive setup. The statistical results comparing the two gestures are shown in Fig 9. In the upper left plot of the figure we examine the forces applied to the tool ($\bar{F^{t}}$), the forces applied to the workpiece ($\bar{F^{c}}$) as well as the shear force ($\bar{F_{s}}$). The plot to the right shows the toque at sensor position ($\bar{T^{t}}$) and the hand torque ($\bar{T_{h}}$). On the lower left side stroke length ($\bar{\Delta X}$) and total stroke length ($\bar{\Delta X_{tot}}$) are shown. The lower right plot shows the variance of the *yaw*, *pitch* and *roll* angles.

Force on the tool This is dominated by the *x* and *z* components for both tasks with medians around 10 N for the PT and −40 N for the PA gesture in the *x* direction and −20 N for the PT and −14 N for the PA gesture in the *z* direction. The *y* component shows only a median of −6 N for PA and even less for PT. This is because the tool is mainly moved in the *x* and *z* direction of the tool frame. According to the Levene’s Test for the *x* component variability is higher for the PA gesture at the significance level of 0.05 or lower (*p* = 0.015, *w* = 7.0, *df* = 1).
Force in the camera frame A similar pattern can be seen here. The *y* and *z* components are dominant with 15 N for PT and −18 N for PA in the *y* and −18 N for PT and −31 N for PA in the *z* direction. The *x* components are both lower than 5 N in magnitude. This similarity ($\left\langle F_{y}^{t}\right\rangle$ and $\left\langle F_{x}^{c}\right\rangle$ low) is because the subjects were urged to keep the tool perpendicular to the camera (compare stroke length). Like the *x* component in the tool frame, the *y* component in the camera frame shows clearly opposite signs due to the opposite direction of motion for the PT and PA gestures. The Levene’s Test for the *y* component showed higher variability in PA at the significance level of 0.1 or lower (*p* = 0.05, *w* = 4.24, *df* = 1).
Shear force By definition the shear force is positive for both gestures. For the PA gesture the median (18 N) is slightly higher than for the PT gesture (17 N). According to the Levene’s Test variability is also higher for the PA gesture at the significance level of 0.1 or lower (*p* = 0.06, *w* = 4.03, *df* = 1).
Torque The torque at the location of the force sensor only shows considerable magnitude in *y* direction. Both medians lie around 11 Nm and the variance for PA is higher at the significance level of 0.1 or lower (*p* = 0.06, *w* = 3.86, *df* = 1). The median around 0.3 Nm for PA of *z* could be attributed to an offset of the distally hafted tool tip from the axis through the center of the force sensor along the haft. The hand torque is clearly higher for PA, with a median of approx. 3 Nm compared to 2.2 Nm for PT. Here again, variability is higher for PA at the significance level of 0.05 or lower (*p* = 0.01, *w* = 7.14, *df* = 1).
Stroke length The lower left plot of Fig 9 compares stroke length between the two gestures. The low values for *x* and *z* show that the stroke was mainly pushed/pulled in *y* direction of the camera frame. The maximum of the total stroke length is found in PT with 0.55 m. The medians for both gestures lie around 0.25 m. Results for the total stroke length are almost identical due to the low magnitudes in x and z components.
Orientation The variation in orientation is depicted in the lower right plot of Fig 9. The median of the *roll* angle is 19 deg^2^ for PT and 17 deg^2^ for PA. For the *pitch* angle we see a median for PT of around 15 deg^2^ and 32 deg^2^ for PA. Maximum variation in orientation also occurs for PA in *pitch* angle with 65 deg^2^. At *yaw* angle the median for PT is 21 deg^2^ and for PA around 11 deg^2^.

Using the insights gained from these experiments, we can begin to formulate hypotheses about the expected patterns of wear formation, given the gesture utilized to operate the tool. In general, it can be said that, for most benchmarks which show differences between the experiments ($F_{x}^{t}$, $F_{y}^{c}$, $\bar{F_{s}}$, $T_{y}^{t}$, $\bar{T_{h}}$, *Var*(*pitch*)), the within-group variation for the PA is higher than the corresponding variance for the PT gesture. This is probably due to inherent difficulties in stabilizing the tool while pushing, while at the same time not damaging the hide.

Regarding the working angles, the variance of orientation in the *pitch* angle is clearly higher for the PA gesture, whereas the variance in *yaw* angle is higher for the PT gesture. The *pitch* angle associated with PA is quite variable. This possibly affects the location of wear formation on the working edge, as well as the distribution of the intensity of the wear, with lateral parts being subject to circular movements in the *x*-*y* plane.

The PA gesture also distributes a higher force in the *x* direction of the tool frame, while the PT gesture generates higher forces in the *z* direction. The shear forces generated with the PA gesture are mostly higher than those associated with the PT gesture. Assuming that shear force is the main factor responsible for removing tissue from the hide, it means that the PA gesture could be more effective from this point of view. However, this efficiency comes at a higher energetic price: hand torque is significantly higher with the PA than with the PT gesture. Needing a greater hand torque can be tiring to the person carrying out the task. Ethnographically, this issue is tackled in several ways by adjusting the haft type: in the Alaskan case, the so-called ‘pistol-grip’ is carved into the shaft to give better control [20], whereas in the Chukchi (Siberian) case, a long shaft is transversally-operated with two hands, for the same reason [18]. These technical adaptations are also possibly related to the need to solve the problem of instability associated with the PA gesture.

## 4 Conclusions and Future Work

We have proposed a relatively simple and inexpensive experimental protocol, which gives insight into the dynamic parameters that are known to be relevant for the wear formation process in hafted endscrapers. Exploratory measurement data for two different gestures enable their visualization, characterization, and differentiation using the aforementioned quantitative parameters that play a role in tribological processes. The main conclusion is that the PA gesture shows higher variances for almost all parameters, suggesting that carrying out the task using this gesture requires a great degree of hand control to overcome instabilities. This kind of analysis provides not only quantitative data for modeling wear formation, but also for generating hypotheses about technical solutions, such as the development of the ‘pistol grip’ in Alaskan endscrapers or the two-handed handles known from the Chukchi. Our recording system is easily generalizable and extendable to other tasks and for use with other tools.

Despite the good results delivered by our measurement system, we have identified a number of issues to be addressed in the near future. First, regarding the accuracy of the measurements, we observed some small limitations in the position sensor. If the tool moved faster than 0.9 ms^−1^, the software was not able to locate the tag. This is caused by motion blur in the video image. In order to record faster movements, increased camera frame rate is necessary. We suppose a linear dependency, i.e. for movements twice as fast the frame rate should double. Second, steps in the graph of the tool orientation (mainly yaw and roll angle) occur despite correct detection of the tag. A reason for this might be the failure of the AprilTags algorithms to correct for inaccuracies in tag detection. These problems can be eliminated by improved camera hardware (e.g. global shutter, faster sampling), improved AprilTags software (e.g. implementation of Kalman filter) or by the application of two tags to the tool and subsequent redundant tracking. Third, the accuracy of our system is currently not as high as a comparable dedicated motion tracking system (e.g. OptiTrack, VICON). Therefore, the presented calibration results present preliminary results and an upper (worst-case) bound. The precision depends on many choices such as optics, tag size, etc. Improving the accuracy and establishing the exact limits of such a system is ongoing work. However, a critical advantage of our system in comparison to the dedicated motion capture systems is its affordability. This makes a widespread application and use in research and education in paleoanthropology labs feasible. While not demonstrated here, the setup can be straightforwardly adapted for use outside the lab.

Beside technical improvements to the measurement system, a larger database of technical gestures and their associated dynamical parameters must be built before important conclusions about ancient technical gestures can be reached. Further experiments are needed in order to generate a statistically representative set of tasks carried out by a more diverse group of participants in a larger variety of settings. These include varying the position of the hides, the type of haft (e.g. mastic hafting, such as is typical of *tulas* in Australia [32]), and other factors. Moreover, mechanisms to integrate the sensor components into non-hafted tools must be sought, in order to expand the scope of the possible applications to remoter periods when hafted tools did not exist. Finally, a systematic investigation of the actual wear formation during these experiments is necessary to begin building a database of dynamic parameters and their corresponding wear patterns. Despite the negative findings published recently by Key [10], it is possible that a more finely-tuned recording setup, such as the one described here, combined with a program of sequential tribological measurements to capture wear accrual might reveal the role of gesture in wear formation.

## Supporting Information

S1 Table Statistical data. This table shows the data used for the statistical analysis. The benchmarks were evaluated for each experiment. Column “task”, “hand” and “workpiece” also show the task, that was performed (PA, PT), which hand the subject used during the experiment (dominant or non-dominant) and the type of hide that was used (fresh or dried). Column “N” gives the number of cycles that were identified in the experiment.
(XLS)

S1 Dataset Measurement data. The zip file contains csv documents of the measurement data generated during the experiments. For each experiment exist two files, one of the measurements of the force sensor and one of the position sensor. They are labeled by the abbreviation “exp” followed by a number and the postfix “-force” for the force sensor and “-position” for the position sensor. The first row shows the legend of the document which states the arrangement of the quantities below. The quantities are listed in chronological order. Sample times of both sensors were 0.01 s.
(ZIP)
